# Mucosal circadian rhythm pathway genes altered by aging and periodontitis

**DOI:** 10.1371/journal.pone.0275199

**Published:** 2022-12-06

**Authors:** Jeffrey L. Ebersole, Octavio A. Gonzalez

**Affiliations:** 1 Department of Biomedical Sciences, School of Dental Medicine, University of Nevada Las Vegas, Nevada, Nevada Las Vegas; 2 Center for Oral Health Research, College of Dentistry, University of Kentucky, Lexington, Kentucky; 3 Division of Periodontology, College of Dentistry, University of Kentucky, Lexington, Kentucky; University of the Pacific, UNITED STATES

## Abstract

As circadian processes can impact the immune system and are affected by infections and inflammation, this study examined the expression of circadian rhythm genes in periodontitis. **Methods:** *Macaca mulatta* were used with naturally-occurring and ligature-induced periodontitis. Gingival tissue samples were obtained from healthy, diseased, and resolved sites in four groups: young (≤3 years), adolescent (3–7 years), adult (12–26) and aged (18–23 years). Microarrays targeted circadian rhythm (n = 42), inflammation/tissue destruction (n = 11), bone biology (n = 8) and hypoxia pathway (n = 7) genes. **Results:** The expression of many circadian rhythm genes, across functional components of the pathway, was decreased in healthy tissues from younger and aged animals, as well as showing significant decreases with periodontitis. Negative correlations of the circadian rhythm gene levels with inflammatory mediators and tissue destructive/remodeling genes were particularly accentuated in disease. A dominance of positive correlations with hypoxia genes was observed, except HIF1A, that was uniformly negatively correlated in health, disease and resolution. **Conclusions:** The chronic inflammation of periodontitis exhibits an alteration of the circadian rhythm pathway, predominantly via decreased gene expression. Thus, variation in disease expression and the underlying molecular mechanisms of disease may be altered due to changes in regulation of the circadian rhythm pathway functions.

## Introduction

Circadian rhythms are autonomous, self-sustained oscillations in biologic processes entrained to environmental cues [1]. These circadian rhythms are the basis of the concept of chronobiology. These circadian clocks are intimately involved in controlling multiple physiologic and metabolic features on a daily basis in humans and other mammalian species [2–5]. Importantly, these circadian processes also impact the immune system including cell numbers and functions [1,4,6–16]. Triggering of these circadian processes is driven by entrainment actions that focus on the capacity of environmental factors, including light cycle, temperature, and access to nutrition to impact biological clocks [1,3,17,18]. Virtually all cells in the body display peripheral clock activities that coordinate cellular processes, including cells and tissues of the immune system.

The molecular clock cellular machinery is composed of a set of proteins present as two auto-regulatory pathways of transcription and translation [1,2]. The main loop is composed of a positive loop of Circadian Locomoter Output Cycles Kaput (CLOCK), NPAS2, and ARNT-like protein 1 (i.e., BMAL1). These molecules dimerize to control the timing of expression of various genes in the pathway. CLOCK/BMAL1 also regulate transcription of negative controllers in the loop including Period genes (PER1, PER2, PER3) and Cryptochrome genes (CRY1, CRY2) that dimerize and are phosphorylated by casein kinases 1 δ and ε (CSKN1D, CSKN1E) enabling them to translocate to the nucleus and control the CLOCK/BMAL1 complex. Existing data support that 2–10% of mammalian genes are regulated by these circadian clock pathways [18].

Studies of numerous infectious diseases have identified the expression of circadian rhythm factors on susceptibility and severity to these infections [5,15,19–23]. Additionally, chronic inflammatory diseases are associated with disruptions in circadian rhythm entrainment and pathway functions [5,6,8,24,25]. Thus, as periodontitis represents a global chronic inflammatory disease, understanding circadian patterns of immune responses and how the circadian rhythm genes interface with tissue regulatory genes and responses at mucosal tissues in the oral cavity would be expected to reflect important host components of health and disease [26].

## Methods

### Animals and diet

Rhesus monkeys (*Macaca mulatta*) (n = 34; 20 male, 14 female) housed at the Caribbean Primate Research Center at Sabana Seca, Puerto Rico were examined for periodontal health and naturally-occurring disease [27–29]. This included 6 groups: healthy young (≤3 years; n = 5); healthy adolescent (3–7 years; n = 5); healthy adult (12–16 years; n = 7); healthy aged (18–23 years; n = 6); periodontitis adult (12–16 years; n = 5) and periodontitis aged (18–23 years; n = 6). As an estimate, 1 monkey year approximates 3.5 human years, thus the groups represented human subjects about 6–9 yo, 11–25 yo, 42–56 yo, 63–82 yo. A second cohort (n = 36; 17 male, 19 female) was used to determine the results from a ligature-induced periodontitis model. Young, adolescent, adult and aged animals with the same age distribution were used in the study with 9 animals/group with husbandry practices as we have reported previously [30–32] and approved by the Institutional Animal Care and Use Committee (IACUC) of the University of Puerto Rico [33]. The animals are raised in large corrals of family units, and, thus, the housing and environmental enrichment is accomplished via daily interactions of the large group of animals in the corrals with climbing and play opportunities and are rather consistent throughout the study sample interval. Feeding is on a standard daily timeframe using pelleted chow with a 20% protein, 5% fat, and 10% fiber commercial monkey diet (diet 8773, Teklad NIB primate diet modified: Harlan Teklad). The diet is supplemented with fruits and vegetables, and water is provided *ad libitum* in an enclosed corral setting. As such, related to circadian rhythms, the environmental entrainment of the animals is quite similar due to the husbandry employed in the primate facility. The oral sampling procedure using IV sedation of the animals with a standard nonhuman primate cocktail, generally to sedate the animals for approximately 45 min. for the oral exam and procedures. No analgesia is required since the procedures are not considered painful within USPHS guidelines determined by the veterinary staff. No animals are euthanized in this protocol, and all animals are returned to their family units and corrals after each sampling and at the completion of the study. As these animals are housed in large family unit groups, standard examination of the overall population is made on a daily basis by the animal care staff. This includes assessing eating behaviors, activity, and interaction within the family unit for each of the animals.

The clinical examination included probing pocket depth (PPD) and bleeding on probing (BOP; 0–5 scale) [34]. Periodontal health was defined by mean Pocket Depth (PD) ≤ 3.0 mm and mean Bleeding on Probing (BOP) ≤ 1 (0–5 scale) in a full mouth examination excluding 3^rd^ molars and canines [33]. Ligature-induced periodontal disease was initiated and gingival and subgingival plaque samples taken at 0.5, 1, and 3 months (Initiation/Progression), and 2 months after removal of ligatures (Resolution). Ligated teeth included the 1^st^ premolar and 1^st^ and 2^nd^ molars in each of the four quadrants. Determination of periodontal disease at the sampled site was documented by assessment of the presence of BOP and probing pocket depth of >4 mm, as we have described previously [28]. Each animal provided a gingival tissue sample at baseline, during the three disease times, and at resolution. All of these were obtained from distinct unique sites at each time point. In the ligation model, differences in BOP and PPD in health, during disease initiation and progression, and with resolution in these age groups have been described previously [35]. All animals demonstrated significant increases in BOP within 0.5 months (0.6±0.1 to 3.8±0.1; mean±SEM), with somewhat elevated levels in the younger age groups. PPD increases at ligated teeth were also seen in all animals across all age groups with peak disease at 1–3 months. Both young and adolescent animals showed PPD measures of disease that were less than in the adult and aged groups (young/adolescent: 1.4±0.1 to 3.9±0.2; adult/aged: 2.7±0.1 to 5.3±0.2). The clinical resolution of the sites occurred over 2 months resulting only from removal of the ligatures. At resolution, both BOP and PPD measures decreased across all age groups, albeit generally remaining above measures for the baseline, healthy tissues.

### Gingival tissue sample collection and mRNA analysis

Gingival tissue samples from healthy and diseased sites were surgically collected using a standard gingivectomy technique (eg. crevicular incision followed by an interdental incision at the base of the papillae) providing a gingival sample from each animal at each time point and total RNA was extracted for microarray analysis [28]. All samples were collected from the animals between 8:30–11 AM following an overnight NPO requirement for anesthesia. With the naturally-occurring disease model, one gingival biopsy was collected from either a healthy site or periodontal disease site from each animal. In the longitudinal study, each animal provided a gingival tissue sample at baseline, during the three disease times, and at resolution. All of these were obtained from distinct unique sites at each time point. The GeneChip® Rhesus Macaque Genome Array (Affymetrix) was used for the aging health and naturally-occurring periodontitis study and the GeneChip® Rhesus Gene 1.0 ST Array (Affymetrix, Santa Clara, CA, USA) for the ligature-induced periodontitis model, similar to methods we have described previously [28,36–39]. The data were only compared within samples using each type of gene chip, and not combined for the analyses.

Based upon the microarray outcomes we selected 4 genes and performed a qPCR analysis using a standard technique in our laboratory [33,38]. Primers were prepared for RORA (forward—CTATCCCTCCAAGGCACAAG; reverse–AACACAAGACTGACGAGCACA; 110 bp), FOS (forward–GCCTCTCTTACTACCACTCACC; reverse–AGATGGCAGTGACCGTGGGAAT; 126 bp), BMAL1/ARNTL (forward—TGCCACCAATCCATACACAG; reverse–TTCCCTCGGTCACATCCTAC; 123 bp), NPAS2 (forward—AACCTCGGCAGCACTTTAAC; reverse–GGTTCTGACATGGCTGTGTG; 118 bp) and GAPDH (forward–GGTGTGAACCATGAGAAGTATGA; reverse–GAGTCCTTCCACGATACCAAAG; 123 bp) designed using software PrimerQuest at Integrated DNA Technologies website (www.idtdna.com) and were synthesized by Integrated DNA Technologies, Inc. (Coralville, IA). The level of message was determined using the CT values of detected genes calculated in relation to GAPDH by the 2^-ΔΔCT^ method and those levels compared across the RNA samples prepared from each of 5 animals in the young and adult age groups.

### Data analysis

Circadian rhythm-associated genes (n = 42) and their correlation with inflammatory, tissue destructive/remodeling, and hypoxia pathway genes were the focus of this analysis (**Table 1**). The expression intensities across the samples were estimated using the Robust Multi-array Average (RMA) algorithm with probe-level quintile normalization, as implemented in the Partek Genomics Suite software version 6.6 (Partek, St. Louis, MO). The age groups were initially compared using one way ANOVA. For genes that had significant mean differences, two sample t-tests were used to investigate differences compared to healthy adult levels of expression (naturally-occurring disease) or to baseline/healthy tissue samples (ligature-induced disease). Statistical significance was considered by an adujsted p-value < 0.05. The data has been uploaded into the ArrayExpress data base (www.ebi.ac.uk) under accession number: E-MTAB-1977 and into GEO accession GSE180588 (https://www.ncbi.nlm.nih.gov/gds). Correlation analyses were determined using a Pearson Correlation Coefficient with a p-value <0.02.

**Table 1:** Pathway genes examined in study.

Gene ID	Gene Name	Fxn Category	Gene ID	Gene Name	Fxn Category
ARNTL/ BMAL1	Aryl Hydrocarbon Receptor Nuclear Translocator Like	TA	IL10	Interleukin-10	AF
ARNTL2	Aryl Hydrocarbon Receptor Nuclear Translocator Like 2	TA	IL1B	Interleukin-1β	PF
BHLHE40	Basic Helix-Loop-Helix Family Member E40	TP	IL4	Interleukin-4	AF
BHLHE41	Basic Helix-Loop-Helix Family Member E41	TP	IL6	Interleukin-6	PF
BTRC	Beta-Transducing Repeat Containing E3 Ubiquitin Protein Ligase	U	TGFB1	Transforming growth factor β1	AF
CIPC	CLOCK Interacting Pacemaker	TR	TNF	Tumor necrosis factor α	PF
CLOCK	Clock Circadian Regulator	TA	CTSK	Cathepsin K	TD/R
COMMD3/ BMI1	COMM Domain Containing 3	TR	MMP14	Matrix metallopeptidase 14	TD/R
CREB1	CAMP Responsive Element Binding Protein 1	TF	MMP2	Matrix metallopeptidase 2	TD/R
CRY1	Cryptochrome Circadian Regulator 1	TR	MMP7	Matrix metallopeptidase 7	TD/R
CRY2	Cryptochrome Circadian Regulator 2	TR	MMP9	Matrix metallopeptidase 9	TD/R
CSNK1D	Casein kinase 1δ	K	BGLAP/OCN	Osteocalcin	BR
CSNK1E	Casein kinase 1ε	K	COL1A1	Collagen type I Alpha 1	BF
DBP	D-Box Binding PAR BZIP Transcription Factor	TF	MKI67	Marker of proliferation Ki-67	BF
DNMT1	DNA Methyltransferase 1	TR	POSTN	Periostin	BF
EZH2/ MLL1	Enhancer Of Zeste 2 Polycomb Repressive Complex 2 Subunit)	TP	RUNX2	Runx family transcription factor 2	BF
FBXL3	F-Box And Leucine Rich Repeat Protein 3	COR	SPP1/OPN	Secreted phosphoprotein 1/ Osteopontin	BF
FBXL21	F-Box And Leucine Rich Repeat Protein 21, Pseudogene	COR	TNFRSF11B/ OPG	Osteoprotegerin	BF
FOS	Fos Proto-Oncogene, AP-1 Transcription Factor Subunit	TF	TNFSF11/ RANKL	Receptor activator of nuclear factor-κB ligand	BR
HDAC3	Histone deacetylase 3	TR	ARNT/ HIF1β	Aryl Hydrocarbon Receptor Nuclear Translocator	HTF
HNF4A	Hepatocyte Nuclear Factor 4 Alpha	TR	COPS5	COP9 Signalosome Subunit 5	HR
ID2	Inhibitor Of DNA Binding 2	TR	EPAS1/ HIF2α	Endothelial PAS Domain Protein 1	HTF
KITLG	Kit ligand	CS/CP	HIF1A	Hypoxia Inducible Factor 1 Subunit Alpha	HTF
MIR155HG	MicroRNA 155	TR	HIF1AN	Hypoxia Inducible Factor 1 Subunit Alpha Inhibitor	HR
NFE2L2	Nuclear Factor, Erythroid 2 Like 2	TF	HIF3A	Hypoxia Inducible Factor 3 Subunit Alpha	HR
NFIL3	Nuclear Factor, Interleukin 3 Regulated	TF	NCOA1	Nuclear receptor coactivator 1	HR
NPAS2	Neuronal PAS Domain Protein 2	TA			
NR1D1	Nuclear Receptor Subfamily 1 Group D Member 1	TP			
NR1D2	Nuclear Receptor Subfamily 1 Group D Member 2	TP			
PER1	Period Circadian Regulator 1	TR			
PER2	Period Circadian Regulator 2	TR			
PER3	Period Circadian Regulator 3	TR			
PRKAA1/ AMPK	Protein Kinase AMP-Activated Catalytic Subunit Alpha 1	K			
PTEN	Phosphatase And Tensin Homolog	CS/CP			
RICTOR	RPTOR Independent Companion Of MTOR Complex 2	CS/CP			
ROMO1	Reactive Oxygen Species Modulator 1	CS/CP			
RORA	RAR Related Orphan Receptor A	NR			
RORB	RAR Related Orphan Receptor B	NR			
RORC	RAR Related Orphan Receptor C	NR			
RPS6KA5/ MSK1	Ribosomal Protein S6 Kinase A5	K			
RPTOR	Regulatory Associated Protein Of MTOR Complex 1	CS/CP			
USP2	Ubiquitin Specific Peptidase 2	U			

Functional category designation: COR–circadian oscillation regulator; CS/CP–cell survival/cell proliferation; K–kinase; NR–nuclear receptor; TA–transcription activator; TF–transcription factor; TP–transcription repressor; TR–transcription regulator; U–ubiquination; AF–anti-inflammatory; PF–pro-inflammatory; TD/R–tissue destruction/remodeling; BR–bone resorption; BF–bone formation; HTF–hypoxia transcription factor; HR–hypoxia regulator.

## Results

### Naturally-occurring periodontitis and circadian rhythm

**Fig 1:**
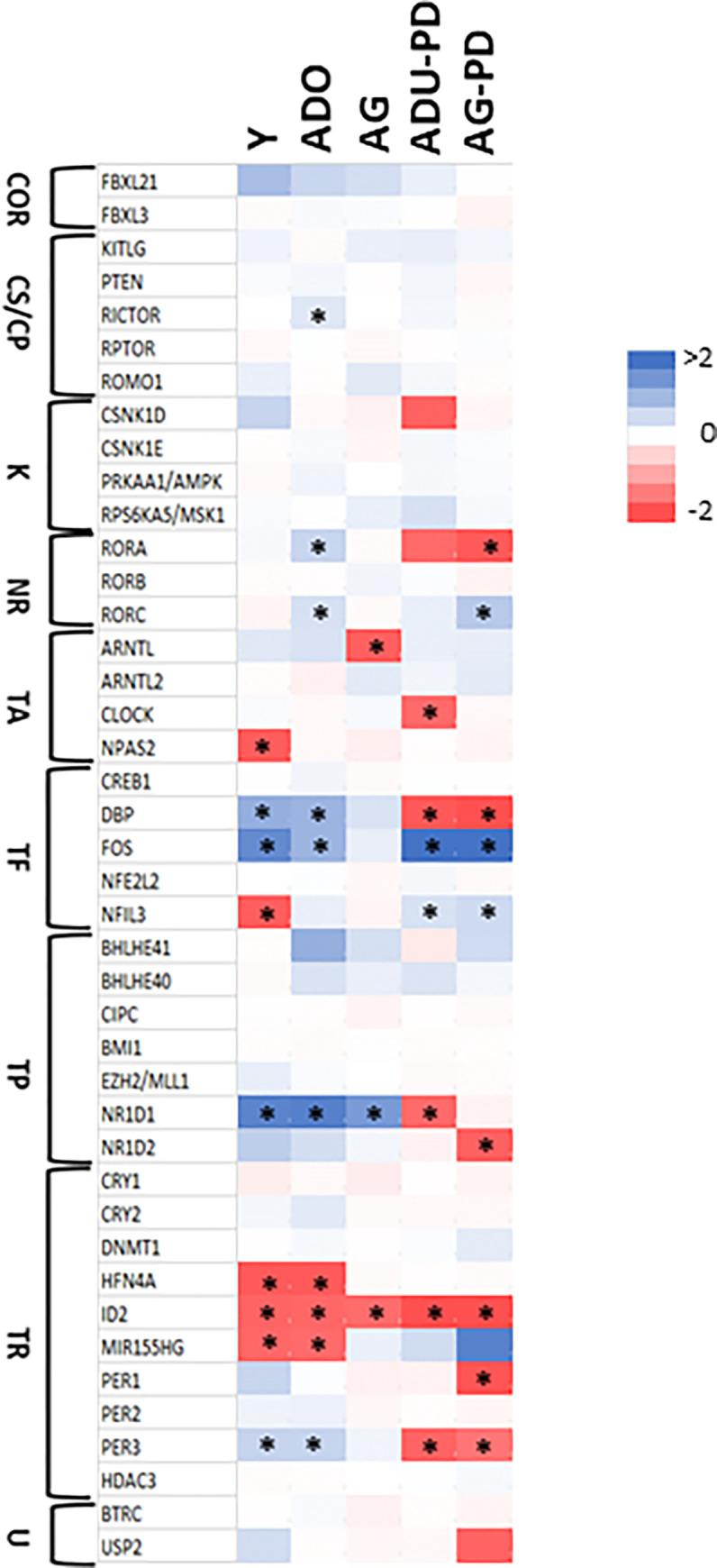
Heatmap of expression of circadian rhythm pathway gene profiles in healthy (Y, ADO, AG) and naturally-occurring periodontitis (ADU-PD, AG-PD) gingival tissues samples. All values are compared as differentially fold-expressed to healthy adult tissues. Functional designation of the genes include: COR–circadian oscillation regulator; CS/CP–cell survival/cell proliferation; K–kinases; NR–nuclear receptor; TA–transcription activator; TF–transcription factor; TP–transcription repressor; TR–transcription regulator; U–ubiquination. Asterisk (*) denotes significantly different from healthy adult levels at p<0.05.

**Fig 1** compares the expression of circadian rhythm pathway genes in health across the lifespan versus healthy adult tissue levels. Young animals showed significantly lower expression of NPAS2, NFIL3, NR1D1, HFN4A, ID2, and MIR155HG, with elevated levels of DBP and FOS. In adolescent tissues, RORA, RORC, DBP, FOS, NR1D1, and PER3 were increased, while HFN4A, ID2 and MIR155HG were decreased similar to the young samples. Aged healthy tissues were generally similar to adult samples albeit ARNTL/BMAL1 and ID2 were both decreased and NR1D1 was increased, as in the other age groups.

Circadian gene expression in naturally-occurring periodontitis in adult and aged animals is also hown in **Fig 1**. Naturally-occurring periodontitis is rarely seen in young/adolescent nonhuman primates, similar to humans. Periodontitis in adults resulted in decreased levels of CSNK1D, RORA, CLOCK, DBP, NR1D1, ID2, and PER3 and a substantial increase in expression of FOS. Aged diseased tissues exhibited similar decreases in expression of RORA, DBP, and PER3 together with NR1D2, PER1, and USP2 that were only affected in the aged samples. Increased expression of RORC, FOS, NFIL3, and MIR155HG was limited to the aged tissues.

### Experimental ligature-induced periodontitis and circadian rhythm

**Fig 2:**
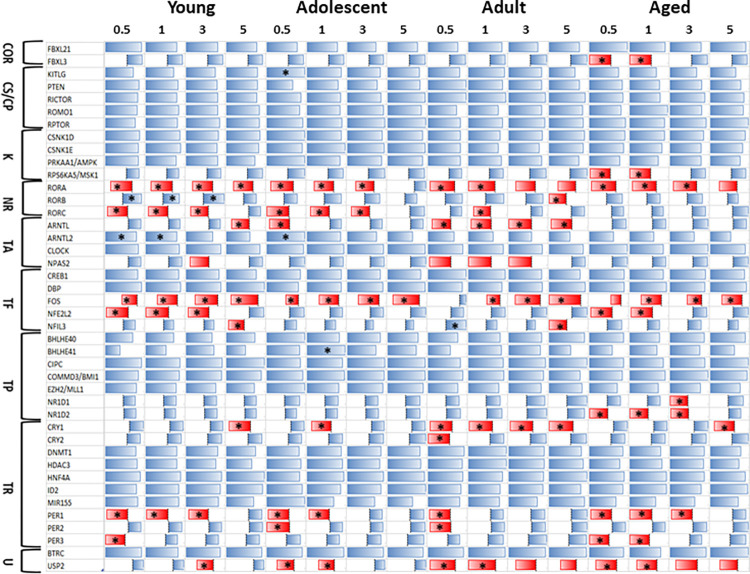
Circadian rhythm pathway gene expression profiles in young, adolescent, adult, and aged gingival tissues samples during ligature induced disease. This analysis used a z-score normalization within a gene across all time points compared to adult baseline (healthy) values. Functional designation of the genes are as in Fig 1. Red bars denote decreased levels and asterisk (*) denotes significantly different from healthy adult levels at p<0.05.

Using the nonhuman primates provided the ability to follow the gingival transcriptome during initiation, progression, and resolution of disease (**Fig 2**). A major decrease in FOS was observed in this early disease model and with resolution in samples from young animals. Similarly, all time points showed decreased expression of RORA. Levels of RORC, NPAS2, NFE2L2, PER1/3, and USP2 were all also decreased with disease. ARNTL, NFIL3, and CRY1 were all decreased in resolution samples. In contrast, only RORB and ARNTL2 were increased with disease. Somewhat similar profiles were observed with the adolescent samples. Beyond the substantial decrease in FOS expression at all time points, RORA, RORC, ARNTL, CRY1, PER1/2, and USP2 were decreased with disease. As with the young samples, ARNTL2 was increased with disease. Adult gene expression patterns are also demonstrated in **Fig 2**. Again, FOS expression was substantially decreased at all time points. RORA, RORC, ARNTL, NPAS2, CRY1/2, PER1/2, and USP2 were all decreased with disease, while RORB and NFIL3 were only decreased in resolution samples, although NFIL3 was elevated early in the disease process. Aged samples also displayed significant decreases in FOS expression at all time points. Also, FBXL3, RPS6KA5, RORA, NFE2L2, NR1D1, NR1D2, PER1/3, and USP2 were all decreased with disease, while CRY1 was only decreased in resolution samples. No circadian pathway genes were up-regulated with disease or resolution in the aged tissue samples.

**Table 2** provides a summary of qPCR validation of 4 genes comparing expression at baseline and 3 months in a subset of young and adult samples. The results demonstrated a general directional agreement in the decreased expression of these genes with disease, and the decrease was somewhat more pronounced in the qPCR analysis.

**Table 2:** qPCR validation of specific circadian related genes at 3 months of disease progression.

Gene ID		Young	Adult
RORA	qPCR	-3.03±3.58	-1.96±0.58
	GeneChip	-1.45±0.44	-1.59±0.43
FOS	qPCR	-3.45±1.90	-2.78±1.70
	GeneChip	-9.09±6.61	-3.45±2.50
BMAL1/	qPCR	-1.54±0.28	-1.72±0.80
ARNTL	GeneChip	-1.23±0.35	-1.30±1.08
NPAS2	qPCR	1.09±0.28	-1.82±0.46
	GeneChip	-1.27±0.30	-1.45±0.65

Values denoted fold-difference from baseline expression with negative value denoting a decreased fold-expression versus baseline for each gene in the young (n = 5) or adult (n = 5) animals. GAPDH was employed as a housekeeping gene for normalization of the results.

### Circadian rhythm gene expression and gingival responses in naturally-occurring periodontitis

**Fig 3:**
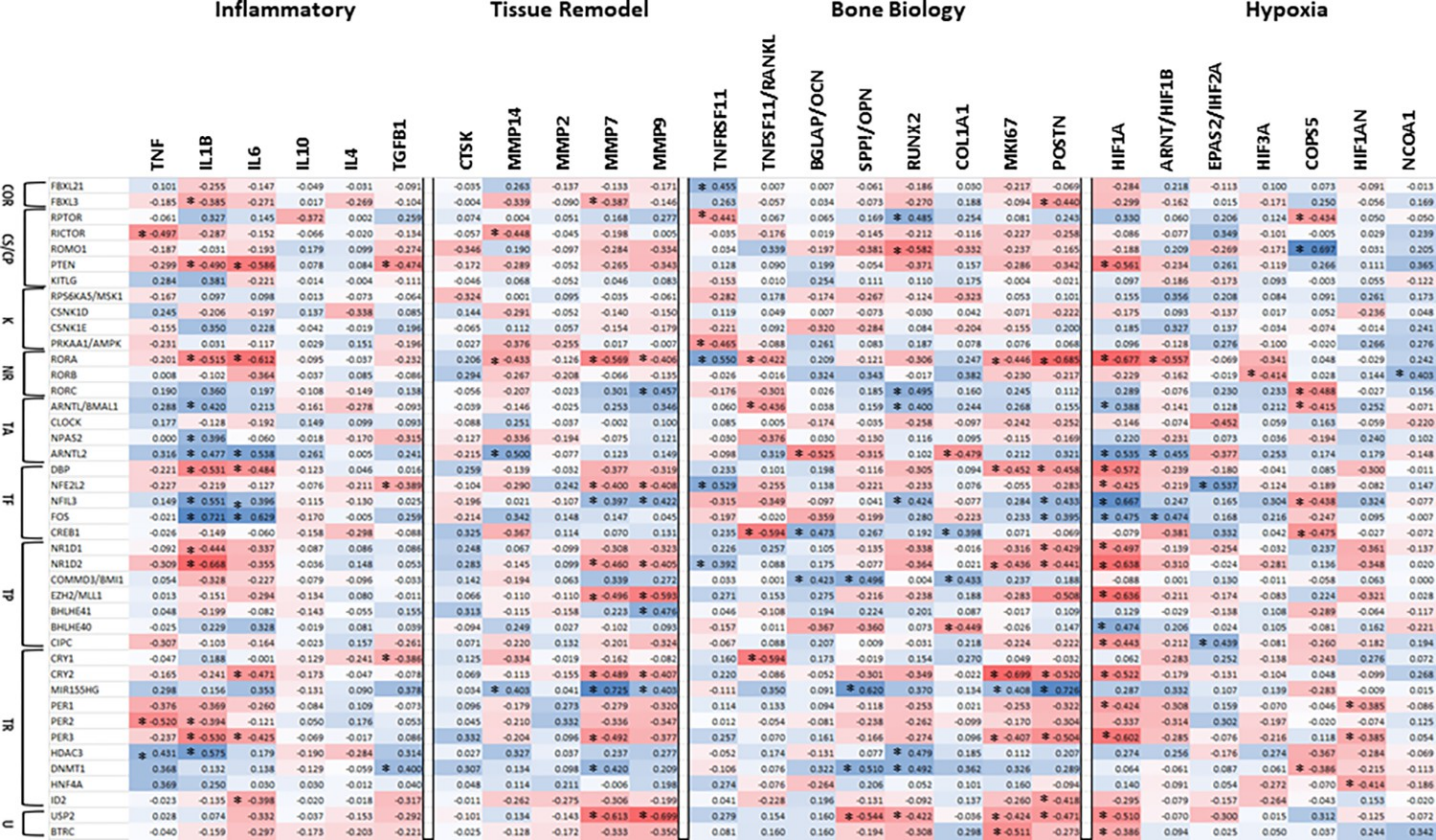
Heatmap of circadian rhythm pathway gene expression profiles correlated with inflammatory genes, tissue destructive/remodeling genes, bone biology genes, and hypoxia genes in gingival tissues from healthy and naturally-occurring periodontitis sites across all age groups. Functional designation of the genes are as in Fig 1. Values denoted by asterisk (*) were significant at p<0.01.

Extensive evidence has identified an array of host factors produced during chronic inflammation that can account for the range of tissue destructive events occurring in periodontitis [40–44]. Thus, these nonhuman primate data were explored for determining interactions between the circadian pathway genes and expression of pro- (TNF, IL1B, IL6) and anti-inflammatory genes (IL4, IL10, TGFB1), as well as genes for products associated with soft tissue destruction or remodeling (CTSK, MMP2, MMP7, MMP9, MMP14) in the cross-sectional study of health and naturally-occurring periodontitis (**Fig 3**). The results showed a specific set of circadian rhythm genes significantly correlated with the inflammatory genes, in particular IL1B and IL6 levels, with individual genes showing either positive or negative correlations. Similar circadian genes were also related to the tissue destructive/remodeling genes, particularly MMP7 and MMP9 showing either positive or negative correlations. Of interest was that there was a general lack of any particular circadian pathway functional category associated with these correlations.

As the integrity of the biology and architecture of alveolar bone is a critical component of periodontitis, we explored the relationship of circadian rhythm genes to gene expression profiles of 8 genes linked to bone functions (**Fig 3**). We noted no particular aspect of the circadian pathway appeared with a tendency towards correlating with the bone genes. However, there did appear to be a predilection of a subset of the bone genes demonstrating either positive or negative correlations with circadian rhythm genes, ranking in frequency of significant correlations as POSTN>RUNX2>TNFRSF11B/OPG> TNFSF11/RANKL.

Finally, a crucial feature of the dysbiosis in periodontal lesions is a substantial increase in the proportion of the microbiome that is anaerobic. Additionally, an environment with low oxygen availability would also dramatically impact the gene expression profiles even within commensal bacteria in these microbiomes. Thus, we evaluated if the changes in circadian rhythm genes would interface with gingival gene expression reflecting an hypoxic environment that would be consistent with a chronic inflammatory lesion in these tissues. **Fig 3** summarizes these correlation patterns, with the greatest frequency of correlations among the circadian pathway transcription activators/factors/repressors. Also, the principal hypoxia transcription factor, HIF1A, showed the greatest frequency of correlations skewed towards being significantly negatively correlated with the circadian rhythm components.

**Table 3** provides an overview summary of the directional correlations for the circadian pathway genes with the inflammatory, tissue destruction/remodeling, bone biology and hypoxia genes in naturally-occurring periodontitis. Notable was the frequency of correlations with RORA and the genes related to inflammation, tissue disruption, and hypoxia, as well as the relationship of levels of HIF1A expression to a broad array of the circadian rhythm genes.

**Table 3:** Significant correlations of circadian rhythm genes with inflammation, tissue destructive/remodeling, bone biology, and hypoxia genes from cross-sectional study in healthy and naturally-occurring periodontitis gingival samples.

	F B L X 2 1	F B X L 3	R P T O R	R I C T O R	R O M O 1	P T E N	P R K A A 1	R O R A	R O R B	R O R C	A R N T L	C L O C K	N P A S 2	A R N T L 2	D B P	N F E 2 L 2	N F I L 3	F O S	C R E B 1	N R 1 D 1	N R 1 D 2	B M I 1	E H Z 2	B H L H E 4 1	B H L H E 4 0	C I P C	C R Y 1	C R Y 2	M I R 1 5 5	P E R 1	P E R 2	P E R 3	H D A C 3	D N M T 1	I D 2	U S P 2	B T R C
**TNF**																																					
**IL1B**																																					
**IL6**																																					
**IL10**																																					
**IL4**																																					
**TGFB1**																																					
**CTSK**																																					
**MMP14**																																					
**MMP2**																																					
**MMP7**																																					
**MMP9**																																					
**TNFRSF11B/OPG**																																					
**TNFSF11/RANKL**																																					
**BGLAP/OCN**																																					
**SPP1/OPN**																																					
**RUNX2**																																					
**COL1A1**																																					
**MKI67**																																					
**POSTN**																																					
**HIF1A**																																					
**ARNT/HIF1B**																																					
**EPAS1/HIF2A**																																					
**HIF3A**																																					
**COPS5**																																					
**HIF1AN**																																					
**NCOA1**																																					

Red highlight denotes a significant negative correlation (p<0.02) and green highlight denotes a significant positive correlation (p<0.02).

### Circadian rhythm gene expression related to inflammatory and tissue destructive/remodeling genes

**Fig 4:**
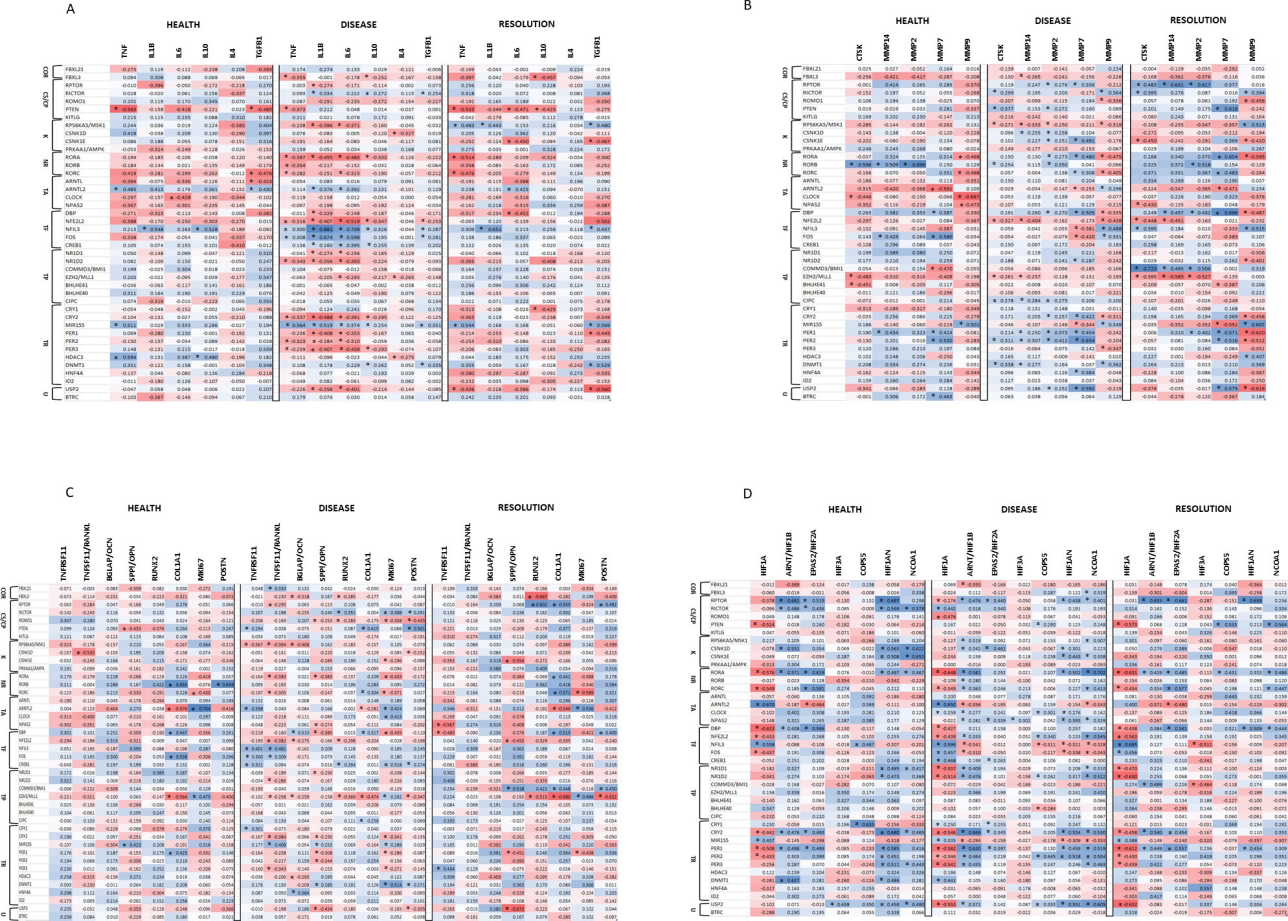
Circadian rhythm pathway gene expression profiles in health, disease, or resolution samples across all age groups correlated with inflammatory genes (**A**), tissue destructive/remodeling genes (**B**), bone biology genes (**C**), and hypoxia genes (**D**). Functional designation of the genes are as in Fig 1. Values denoted by asterisk (*) were significant at p<0.01.

A similar assessment was made regarding circadian rhythm genes in health, disease, and resolution samples of ligature-induced periodontitis. For the inflammatory mediators (**Fig 4A**) Healthy tissue samples (ie. Baseline) showed rather limited correlations with inflammatory mediators, although there appeared some predilection for a relationship to TGFB1 expression. With disease the frequency of significant correlations with inflammatory mediators increased substantially. Additionally, the majority were negative correlations particularly targeted towards TNF, IL1B, and IL6. However, of note, NFIL3, FOS and MIR155 were significantly positively correlated with the bulk of these mediators. Resolution samples demonstrated a limited number of correlations, with TNF and TGFB1 most dominant and circadian representing genes, PTEN and USP2, exhibiting an elevated frequency of these correlations.

While a limited number of correlations were noted with the tissue destruction/ remodeling genes in healthy samples (**Fig 4B**), MMP7 and MMP9 showed the most frequent correlations, and RORB (positive) and ARNTL2 (negative) circadian genes showed multiple significant correlations. Similar to inflammatory genes, correlations with the tissue destruction/remodeling genes were quite frequent with disease. However, in contrast to the inflammatory genes, the preponderance of these responses were positive correlations particularly related to MMP7 and MMP9, and PER2 was positively correlated with all tissue genes but MMP9. Also, numerous circadian genes were negatively correlated with levels of MMP9 expression, and both RPS6KA5/MSK1 and NFE2L2 genes highly correlated with multiple of these tissue destruction/remodeling genes. In resolution samples, the results differed from the inflammatory gene relationships; many circadian genes demonstrated significant correlations with the tissue destruction/remodeling genes. These were enriched for correlations with MMP7, MMP9, and MMP2. Interestingly, RPTOR and BMI1 were positively correlated and EZH2 was negatively correlated with the set of CTSK, MMP14, and MMP2 genes.

### Circadian rhythm gene expression related to bone biology genes

Bone resorptive processes are a hallmark of periodontitis being driven by heightened numbers and activity of osteoclasts [45–49]. In healthy samples from the animals, very few of the bone biology genes were robustly correlated with the circadian pathway genes (**Fig 4C**). These relationships were increased during disease with a wide array of circadian genes significantly correlated with the bone biology genes. However, relative to general functional categorization of the circadian genes there did not appear to be a particular functional category that dominated. In resolution samples, a number of the circadian genes showed individualized correlations with bone biology genes; however, there was no apparent predilection for these interactions.

### Circadian rhythm gene expression related to hypoxia pathway genes

The transition from a symbiotic to dysbiotic microbiome with periodontitis clearly reflects an increasing composition of anaerobic bacteria, supporting the hypoxic nature of the lesions [43,50]. Moreover, chronic inflammation has also been shown to result in tissue hypoxia, thus, examination of the potential role of circadian genes related to these hypoxic changes was explored. In health (**Fig 4D**) there was a large array of correlations between circadian and hypoxia-related genes, with the majority of these being significant positive correlations. Of particular note was the extensive negative correlations between circadian genes and the major hypoxia transcription factor, HIF1A. This pattern was maintained with disease, although an increased number of circadian genes now showed significant positive correlations with HIF1A. The frequency of the correlations decreased in resolution samples; however, HIF1A remained negatively correlated with numerous circadian genes.

**Table 4** provides a summary of these relationships of the circadian pathway genes and these localized biological function genes in the gingival tissues. With inflammatory and tissue destructive/remodeling genes, a dominant number of these correlations were observed with disease, while fewer were noted in health, with resolution samples trending towards the frequency seen in diseased samples, in particular related to MMP7 and MMP9. A high frequency of these correlations were found with the circadian rhythm genes, MSK1, RORA, NFE2L2, NFIL3, FOS, CRY2, MIR155, and PER2. Additionally, TNF, IL1B, IL6, MMP2, MMP7 and MMP9 demonstrated a broad array of correlations across the circadian genes.

**Table 4:** Significant correlations of circadian rhythm genes with inflammation, tissue destructive/remodeling, bone biology, and hypoxia genes from longitudinal study of ligature-induced periodontitis, healthy, and resolution gingival samples.

**HEALTH**																																								
	FBXL21	FBXL3	RPTOR	RICTOR	ROMO1	PTEN	MSK1	CSNK1D	CSNK1E	RORA	RORB	RORC	ARNTL	ARNTL2	CLOCK	NPAS2	DBP	NFE2L2	NFIL3	FOS	CREB1	NR1D1	NR1D2	BMI1	EZH2	BHLHE40	BHLHE41	CIPC	CRY1	CRY2	MIR155	PER1	PER2	PER3	HDAC3	DNMT1	HNF4A	ID2	USP2	BTRC
**TNF**																																								
**IL1B**																																								
**IL6**																																								
**IL10**																																								
**IL4**																																								
**TGFB1**																																								
**CTSK**																																								
**MMP14**																																								
**MMP2**																																								
**MMP7**																																								
**MMP9**																																								
**TNFRSF11B/OPG**																																								
**TNFSF11/RANKL**																																								
**BGLAP/OCN**																																								
**SPP1/OPN**																																								
**RUNX2**																																								
**COL1A1**																																								
**MKI67**																																								
**POSTN**																																								
**HIF1A**																																								
**ARNT/HIF1B**																																								
**EPAS1/HIF2A**																																								
**HIF3A**																																								
**COPS5**																																								
**HIF1AN**																																								
**NCOA1**																																								
**DISEASE**																																								
	FBXL21	FBXL3	RPTOR	RICTOR	ROMO1	PTEN	MSK1	CSNK1D	CSNK1E	RORA	RORB	RORC	ARNTL	ARNTL2	CLOCK	NPAS2	DBP	NFE2L2	NFIL3	FOS	CREB1	NR1D1	NR1D2	BMI1	EZH2	BHLHE40	BHLHE41	CIPC	CRY1	CRY2	MIR155	PER1	PER2	PER3	HDAC3	DNMT1	HNF4A	ID2	USP2	BTRC
**TNF**																																								
**IL1B**																																								
**IL6**																																								
**IL10**																																								
**IL4**																																								
**TGFB1**																																								
**CTSK**																																								
**MMP14**																																								
**MMP2**																																								
**MMP7**																																								
**MMP9**																																								
**TNFRSF11B/OPG**																																								
**TNFSF11/RANKL**																																								
**BGLAP/OCN**																																								
**SPP1/OPN**																																								
**RUNX2**																																								
**COL1A1**																																								
**MKI67**																																								
**POSTN**																																								
**HIF1A**																																								
**ARNT/HIF1B**																																								
**EPAS1/HIF2A**																																								
**HIF3A**																																								
**COPS5**																																								
**HIF1AN**																																								
**NCOA1**																																								
**RESOLUTION**																																								
	FBXL21	FBXL3	RPTOR	RICTOR	ROMO1	PTEN	MSK1	CSNK1D	CSNK1E	PRKAA1	RORA	RORB	RORC	ARNTL	ARNTL2	CLOCK	NPAS2	DBP	NFE2L2	NFIL3	FOS	CREB1	NR1D1	NR1D2	BMI1	EZH2	BHLHE40	BHLHE41	CRY1	CRY2	MIR155	PER1	PER2	PER3	HDAC3	DNMT1	HNF4A	ID2	USP2	BTRC
**TNF**																																								
**IL1B**																																								
**IL6**																																								
**IL10**																																								
**IL4**																																								
**TGFB1**																																								
**CTSK**																																								
**MMP14**																																								
**MMP2**																																								
**MMP7**																																								
**MMP9**																																								
**TNFRSF11B/OPG**																																								
**TNFSF11/RANKL**																																								
**BGLAP/OCN**																																								
**SPP1/OPN**																																								
**RUNX2**																																								
**COL1A1**																																								
**MKI67**																																								
**POSTN**																																								
**HIF1A**																																								
**ARNT/HIF1B**																																								
**EPAS1/HIF2A**																																								
**HIF3A**																																								
**COPS5**																																								
**HIF1AN**																																								
**NCOA1**																																								

Red highlight denotes a significant negative correlation (p<0.02) and green highlight denotes a significant positive correlation (p<0.02).

With respect to the bone biology genes, there was a limited array of interactions in healthy tissues (**Table 4**). With disease, RANKL, MKI67, and POSTN demonstrated numerous correlations with the circadian genes, with RICTOR, DBP, CREB1, and EZH2 showing association with multiple bone biology genes. In resolution samples, numerous correlations with the circadian genes were shown with SPP1/OPN, COL1A1, MKI67, and POSTN bone biology genes. Only DBP and EZH2 circadian genes exhibited frequent correlations with the bone biology genes with disease resolution. Additionally there was substantial overlap in the repertoire of circadian genes demonstrating multiple correlations with the bone biology genes, including RORA and PER1 in health, RPTOR, RORA, DBP, NR1D2, PER1, and USP2 in disease, and RORA and DBP in resolution samples.

In health and disease, except for HIF1A, virtually all of the correlations between circadian and hypoxia genes were positive (**Table 4**). With disease, the circadian genes RPTOR, RORA, DBP, EZH2, MIR155, PER2, PER3 and USP2 were all enriched for these correlations. Interestingy, both FOS and PER demonstrated somewhat unique patterns of negative correlations with an array of the hypoxia genes, except for HIF1A that was positively correlated with these circadian genes. Resolution samples showed more limited relationships for circadian and hypoxia genes; however, the pattern with negative correlations with HIF1A remained. Additionally, RORA was unique in showing numerous correlations.

### Circadian gene interactome

**Fig 5:**
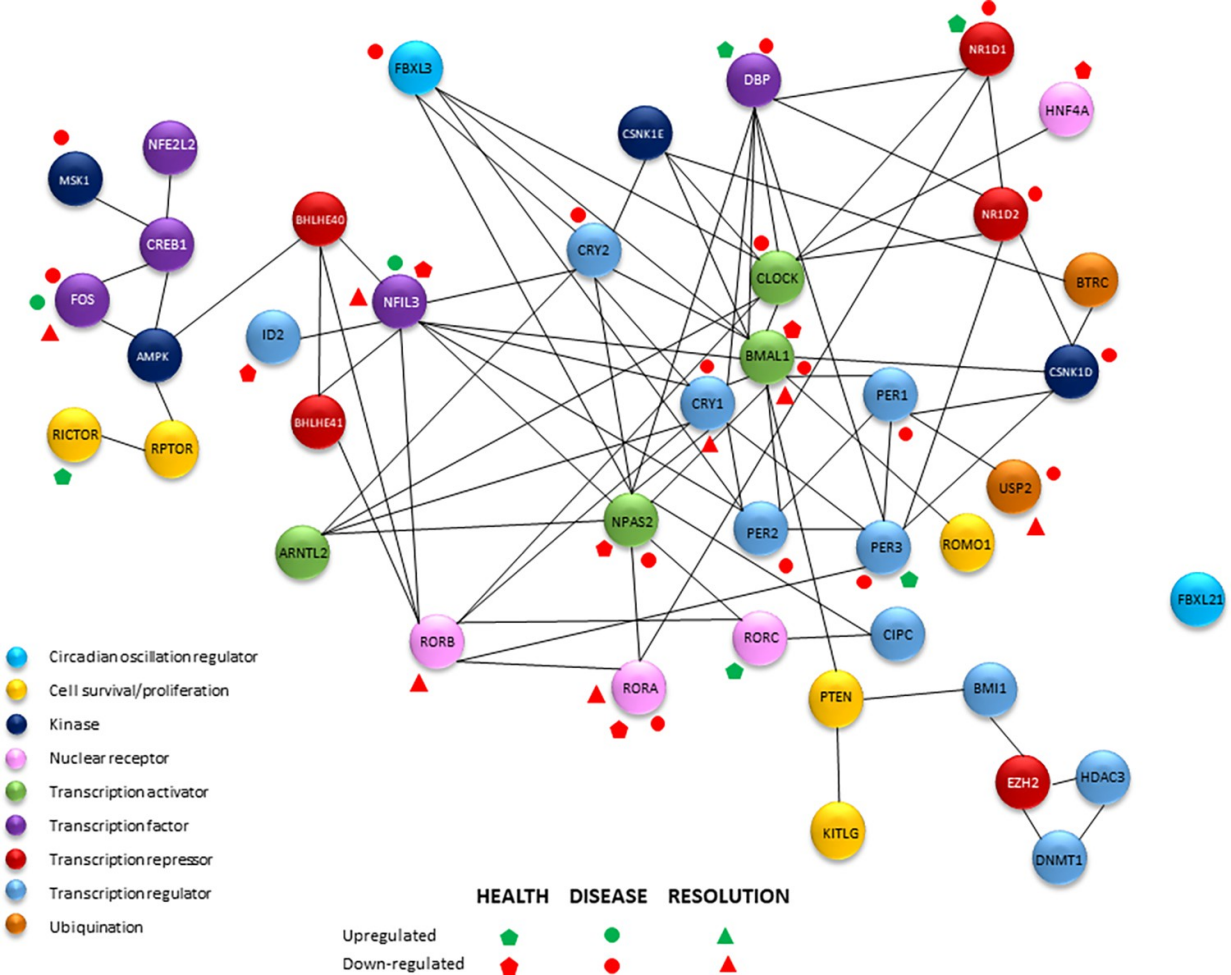
Schematic of interactome of circadian rhythm and circadian-associated genes denoted by black lines connecting the nodes. Colored nodes denote functional categorization of each gene. Green and red symbols denote significant differences in expression in healthy samples (young/adolescent/aged versus adult) or comparing naturally-occurring disease (versus health) and ligature-induced disease (versus baseline healthy samples).

**Fig 5** provides a schematic of the potential circadian gene interactome within the healthy and diseased gingival tissues. The first notable observation is that the majority of differential expression of these genes in healthy tissues of both younger and older individuals was decreased (down-regulated) compared to adult levels. Additionally, longitudinal changes in expression with disease were also generally decreased compared to baseline healthy tissues. Comparison of levels of the circadian pathway genes did appear to identify substantial differences in ARNT/BMAL1, FOS, NFIL3, and RORA that varied in healthy and diseased tissues.

## Discussion

While it is clear that aspects of the oral microbiome trigger a dysregulation of host responses resulting in periodontal lesions, data are still lacking regarding the detailed molecular features of these processes that dictate the initiation, progression and resolution of an individual lesion. An array of host response characteristics [28,40,51–62], which might be predicted to be affected with disease, have been identified with disease. However, some unexpected response profiles and gene polymorphisms [63–69] have been observed with disease. This report describes results of another novel pathway, circadian rhythm, which is altered with both aging and periodontitis.

Inflammation can affect circadian clock pathways [1,4,8,10,70], and clock genes affect the development of various chronic inflammatory diseases, as well as increasing their severity [5]. This study examined circadian pathway gene expression and its relationship to genes associated with tissue alterations occurring in disease using a nonhuman primate model of naturally-occurring and ligature-induced periodontitis. Circadian pathway genes showed some differential expression in healthy tissues from young and adolescent animals compared to adult levels. These were primarily represented by genes related to transcription activator, transcription regulator, and transcription factor functions. In contrast, periodontitis tissues showed an array of genes, including kinases and a broad array of transcription-associated genes related to circadian rhythm that were altered, generally decreased, in both adult and aged tissues. This could be of interest related to the clear evidence of increases in prevalence and severity of periodontitis with age. Extensive variation in aging processes occurs across the human population, and contribute to differences in time of onset of age-associated diseases. Within the mucosa, and specifically oral mucosa, that is continually under septic challenge it must be expected that a combination of multiple pathways are necessary for tissue homeostasis, while substantial breakdown in an individual pathway could significantly enhance risk for disease. Thus, the down-regulation of genes of the circadian pathway with aging could reflect one mechanism increasing the risk for disease on older individuals. The data that showed a general down-regulation of genes that would regulate the circadian pathway with periodontitis is consistent with reports on age effects on maintaining normal circadian rhythm processes and the impact of infection and inflammation on these processes. As periodontitis presents both of these features, this relationship might be expected. However, lacking in this cross-sectional model of naturally-occurring disease is insights into the kinetics of the relationship. Thus, does periodontitis drive alterations in cellular circadian rhythm contributing to chronicity, or does an altered regulation of the circadian pathway increase the risk for triggering the initiation and progression of disease. Further studies will need to be developed potentially with small animal models, albeit, the longitudinal experimental periodontitis models in the nonhuman primates provided some additional guidance.

As in naturally-occurring disease, generally the circadian rhythm gene expression was significantly decreased with ligature-induced periodontitis and this was observed in all age groups. Similar genes were affected, and the timing of these changes during disease initiation and progression appeared generally similar, as has been reported in rheumatoid arthritis [1] and in response to microbial stimulation [11,13,71]. Altered regulation of the circadian rhythm pathway can elevate intracellular production of reactive oxygen species [72] and links directly to the mTOR and PI3K signaling processes related to apoptosis and autophagy [73], as well as processes contributing to cellular senescence [74]. As the circadian rhythm pathway demonstrates multiple control levels, components of the negative feedback loop that are up-regulated by CLOCK and ARNT/BMAL1 include PER1/2/3 and CRY1/2, as well as NR1D1/NR1D2 (REV-ERBα/β) [2]. Of particular interest was that many of these specific genes were significantly altered with periodontitis in the gingival tissues and remained affected even with clinical disease resolution that could be predicted to increase the risk for disease progression, and negatively impact biological resolution. Of interest from this model was that many of the changes in circadian rhythm gene expression were noted as early as 2 weeks post-ligature placement during disease initiation in all age groups. These appeared coincident with increases in inflammation (ie. BOP), but generally were noted prior to the increases in destructive disease measures (ie. PPD). These circadian rhythm gene changes were also observed in the younger animals with more minimal disease expression. Additionally, many of these down-regulated responses were maintained in the clinical resolution samples. Each of these observations suggest that altered circadian rhythm pathway functions may presage and contribute to the risk for disease.

Circadian genes contribute to development of B cells and pro-inflammatory responses [1] in response to infection [2]. This pathway genes also interact with the NF-κB transcription processes [75] to regulate a variety of inflammatory cytokines [76,77] and alter levels of various response molecules in inflammatory reactions [78–80]. Studies of circadian rhythm functions in the oral cavity include data with oral bacteria (ie. *P*. *gingivalis*) [81] and outcomes of oral cells [82] showing effects on this pathway. Moreover, circadian genes have been associated with altered bone metabolism and osteoclast functions [82–84]. Finally, some limited information is available regarding circadian gene expression modulated by hypoxia in oral cells [85,86]. Thus, we explored if the circadian rhythm genes specifically related to various host response components that are present in health and disease in the periodontium. We identified various circadian rhythm genes that significantly correlated with the inflammatory genes and tissue destructive/remodeling genes. These generally were negative correlations, albeit some selected genes related to the circadian pathway were significantly positively correlated (NFIL3, FOS, MIR155). With resolution, the circadian rhythm genes demonstrated significant correlations primarily with the tissue destruction/remodeling genes. The frequency of significant correlations of wide array of circadian rhythm genes and those related to bone biology was generally increased during disease progression. A large array of positive correlations between circadian and hypoxia-related genes was observed in healthy tissue samples. However, an extensive frequency of negative correlations occurred between circadian genes and the major hypoxia transcription factor, HIF1A, in health and resolution, while an increased number of circadian genes showed significant positive correlations with HIF1A in disease. Thus, a clear interaction between the circadian rhythm pathway and potential for important regulation of inflammation, tissue destructive molecules, biomolecules of bone biology, and a tissue hypoxia microenvironment affecting the health of the periodontium was apparent. However, one primary interpretation of the data was that the effects on the circadian genes across the age groups with the development of periodontitis was actually quite similar, rather than substantially different, and in disease there was a somewhat distinctive pattern of circadian gene and local tissue biology gene expression. This observation actually differed from a number of our other analyses targeting immune and inflammatory pathways in gingival tissues that demonstrated substantial differences in expression and regulation with age and disease [31,33,37,38,55,87–90]. Thus, the effects on this pathway seemed to be “conserved” in disease.

While it needs to be acknowledged that many of these circadian pathway transcription regulators and factors have overlap with numerous other cellular pathways, including immune response regulation, apoptosis, autophagy, oxidative stress responses, and fundamental control of cellular survival, there exists substantial data showing mechanistic links of circadian clock functions with health and disease [17]. This is the first study to document *in situ* relationships of genes contributing to this critical host pathway to features of the biology of the mucosal gingival tissues. Importantly, experimental manipulations that can be accomplished in this nonhuman primate disease model, limited clear cause-and-effect conclusions regarding the relationship of the circadian rhythm pathway to molecular aspects of periodontitis triggering pathways. However, the interesting features of circadian pathway changes and correlations with local tissue environmental gene expression may help explain disease variation, as well as suggesting the potential for new targets for management of this chronic disease.
